# Supplementary material for: Transcription factor paralogs orchestrate alternative gene regulatory networks by context-dependent cooperation with multiple cofactors
Source: Nat Commun. 2022 Jul 1;13:3808. doi: 10.1038/s41467-022-31501-2 (PMC9249852; doi:10.1038/s41467-022-31501-2)
Supplement: Supplementary file 1 — Supplementary information [file 41467_2022_31501_MOESM1_ESM.pdf]

a

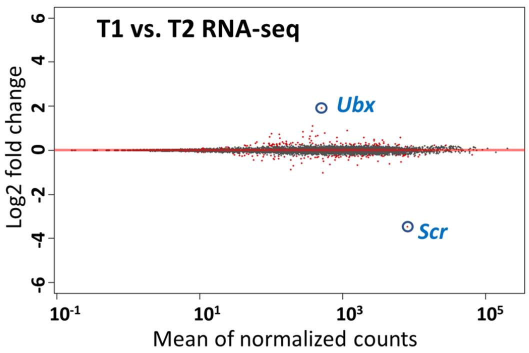

b

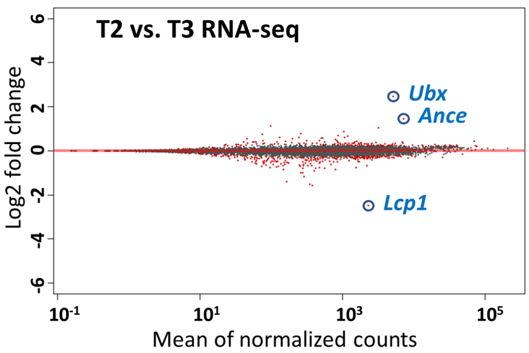

c

- FDR<0.01 AND abs(log2(fold change))>1

		up		
		T1	T2	T3
down	T1		2	8
	T2	2		4
	T3	20	6	

Supplementary Fig. 1

Supplementary Fig. 1. The transcriptomes of the 3 pairs of leg discs are highly similar

a. MA plot comparing the transcriptomes of T1 and T2 leg discs. Differentially expressed genes, which are defined as FDR<0.01, are labeled red.

b. Similar to **a**, except that the comparison is between T2 and T3 leg discs.

c. Table showing the number of differentially expressed genes with at least a 2-fold change for all pair-wise comparisons.

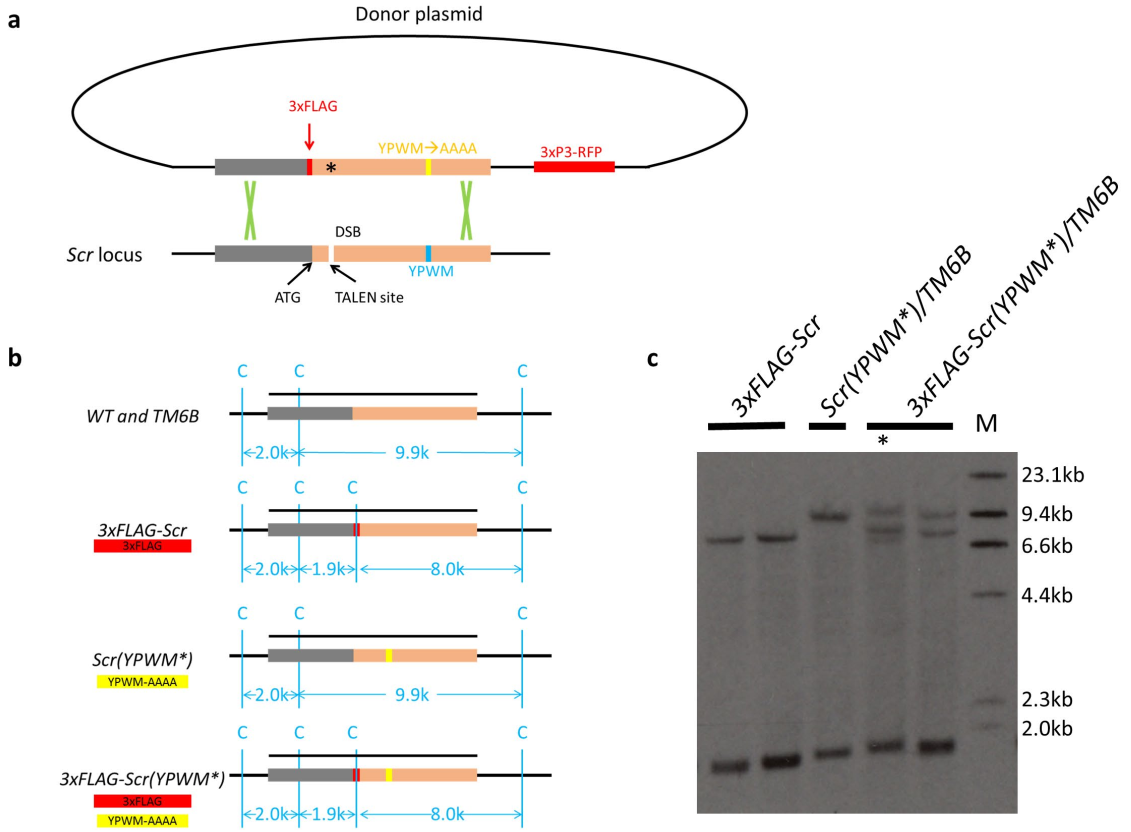

Supplementary Fig. 2

Supplementary Fig. 2. The generation of *3xFLAG-Scr* and *3xFLAG-Scr(YPWM*)* alleles.

a. The strategy used in targeting the *Scr* locus using a pair of TALENs. The orange boxes indicate coding region, and the grey boxes are the 5'UTR. The positions of the ATG start codon, 3xFLAG tag, TALEN target site, the YPWM motif, and the mutated YPWM motif on the donor plasmid are shown. The asterisk indicates that the TALEN target on the donor plasmid is mutated, so the TALENs only cut the genomic DNA. 3xP3-RFP was included on the donor plasmid to select against whole plasmid integration events (see Methods). DSB: double stranded DNA break.

b. ClaI restriction map of various genotypes. The blue lines indicate the ClaI sites relevant to the Southern blot. The positions of the 3xFLAG tag and the YPWM-AAAA mutation are indicated when applicable. The black bars above the genomic loci indicate the fragment used as Southern probe, and the sizes of expected Southern bands are shown.

c. Southern blot result of several alleles obtained in genomic targeting of the *Scr* locus. M: Roche DIG labeled DNA marker II. Asterisk: a potentially erroneous allele with a Southern blot pattern deviated from the expected one.

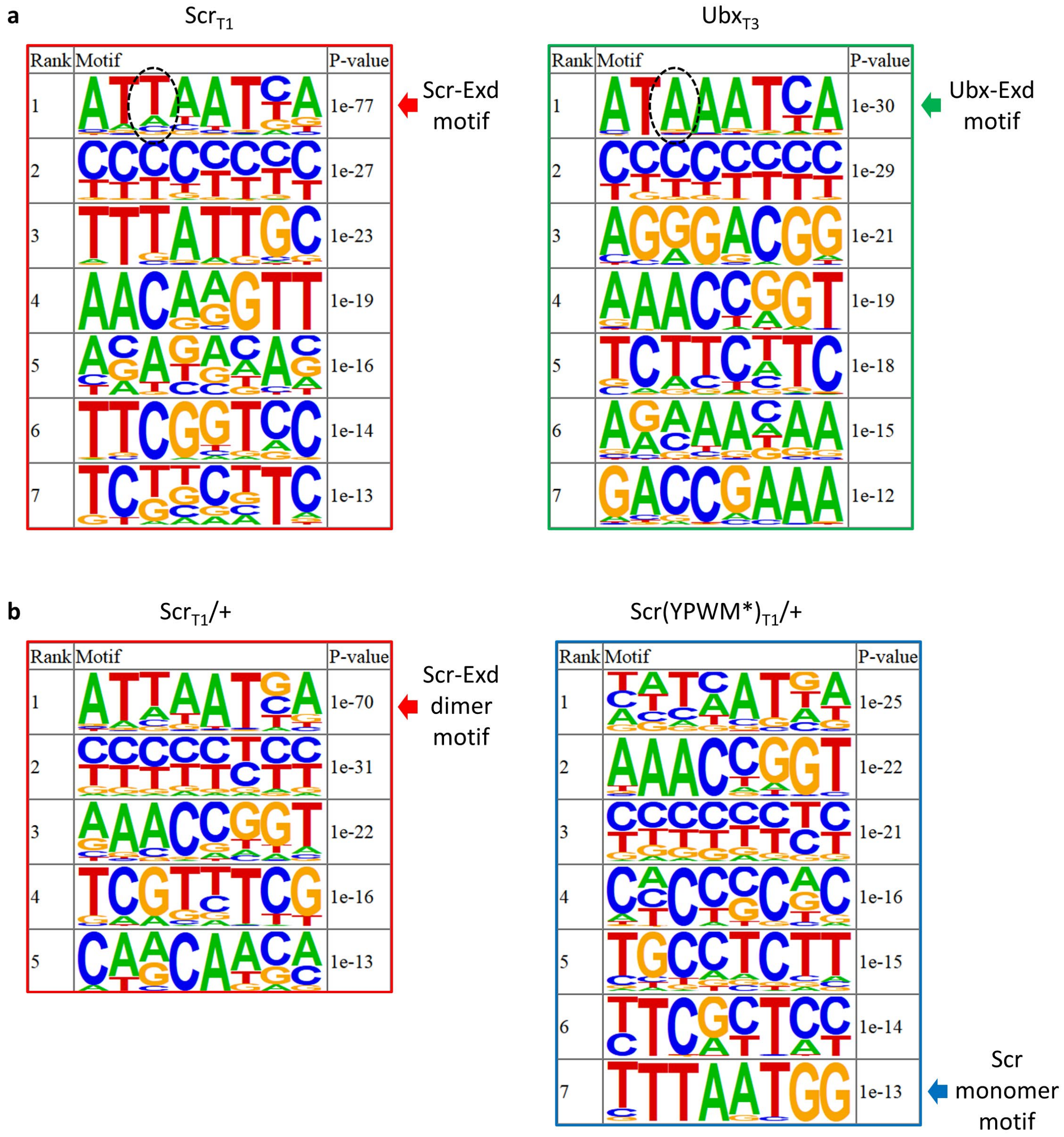

Supplementary Fig. 3

Supplementary Fig. 3. Sequences identified by *de novo* motif searches.

a. Motifs enriched in Scr_{T1} and Ubx_{T3} peaks that are located in intergenic or intronic regions of the genome. Arrows point out known Scr-Exd and Ubx-Exd dimer motifs. Dashed ovals highlight the positions within the Hox-Exd heterodimer motifs that Scr and Ubx are known to have different preferences [3]. Up to 7 significantly enriched motifs are shown.

b. Motifs enriched in Scr_{T1}/+ and Scr(YPWM*)$_{T1}$/+ peaks located in intergenic and intronic regions. Red arrow indicates the known Scr-Exd dimer motif, and blue arrow indicates a potential Scr monomer motif. Up to 7 significantly enriched motifs are shown.

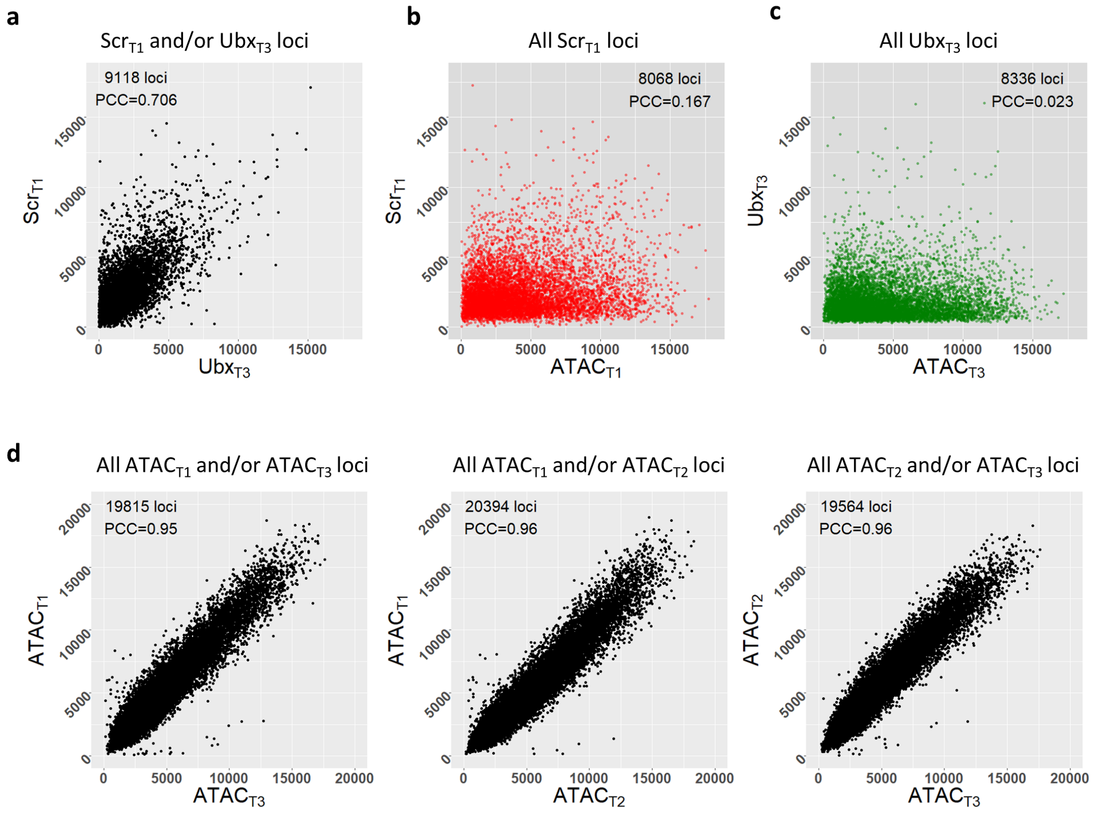

Supplementary Fig. 4

Supplementary Fig. 4. Most paralog-specific Hox bound loci do not show tissue-specific differences in chromatin accessibility.

a. Scatter plot comparing Scr_{T1} and Ubx_{T3} ChIP-seq signals in all Hox bound loci. Hox bound loci are defined as loci bound by either Scr in T1 leg discs or by Ubx in T3 leg discs, or by both (see Methods). The Pearson's correlation coefficient (PCC) between Scr_{T1} and Ubx_{T3} signals in all Hox bound loci is reported.

b and c. Correlation between Scr_{T1} ChIP-seq and $ATAC_{T1}$ signals in all loci bound by Scr in the T1 leg discs (**b**), as well as that between Ubx_{T3} ChIP-seq and $ATAC_{T3}$ signals in all loci bound by Ubx in the T3 leg discs (**c**). The Pearson's correlation coefficient (PCC) is reported.

d. Pair-wise comparisons of ATAC-seq signals between different leg discs. The number of loci and the Pearson's correlation coefficient (PCC) are reported.

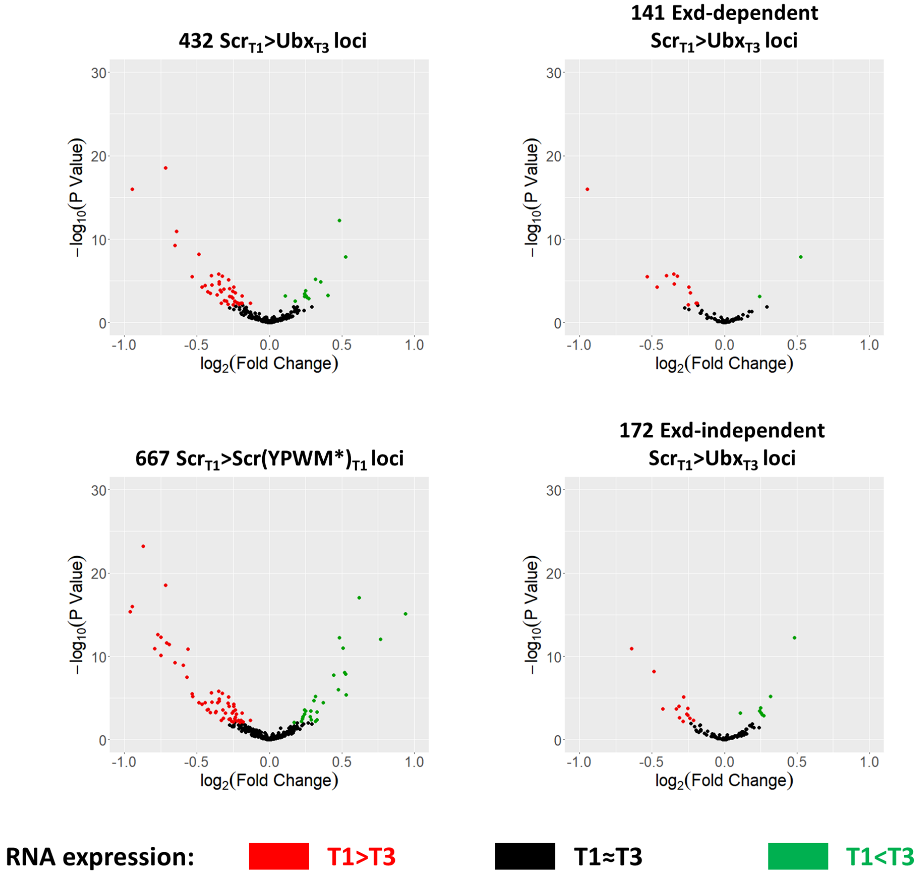

Supplementary Fig. 5

Supplementary Fig. 5. Correlation between Hox-DNA binding and nearby gene transcription.

Volcano plots showing T1 and T3 leg disc RNA expression of genes near different classes of Hox ChIP peaks. Genes with T1>T3 expression have negative log fold change, and those with an FDR<0.05 are colored red. Genes with T1<T3 expression have positive log fold change, and those with an FDR<0.05 are colored green.

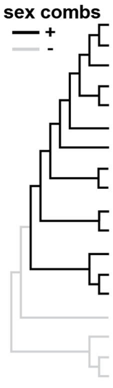


```
mel  CCTTCGGGCACGATTCTTTGCCACTCAATTTATTAAATTTATAAAATATTTATGAAGCCGTCTCTGA---GTACCTTTAC
sim  CCTCCGGGCGCGATTCTTTGCCACTCAATTTATTAAATTTATAAAATATTTATGAAGCCGTCTCTGA---GTACGTTTAC
yak  CCTCCGGGCCAGATTCTTTGCCACTCAATTTATTAAATTTATAAAATATTTATGAAGCCGGCCGAG-----TTGCTGCAA
tak  CCTCCGGGCTGGATTCTTTGCCACTCAATTTATTAAATTTATAAAATATTTATGAAGCCCGCTGAGT---ACGTTTACGT
bia  CCTCCGGGCTGGATTCTTTGCCACCCAATTTATTAAATTTATAAAATATTTATGAAGCCGGCCGAGC---ACGTTTACAC
eug  CCTCCGGGCTGAATTCTTTGCCACTCCATTTAATAAATTTATAAAATATTTATGAAGCCGGCTAAGT---ACGTTTACTT
fic  CCTTCGGGCTGGATTCTTTGCCACTCAATTTATTAAATTTATAAAATATTTATGAAGCCAGCTGAGC---TGAGTTAAGT
ele  CCTCTGGGCTGAATTCTTTGCCACTCCATTTATTAAATTTATTAAATATTTATGA---AGACTAAGT---ACGTTTGCTT
rho  CCTCCGGGCTGAATTCTTTGCCACTCCATTTAATAAATTTATAAAATATTTATGAAGCCGGCTAAGT---ACGTTTACTT
ana  CCCAGCGGCTATATTCTTCGCCACTCAATTTATTAAATTAGTTAAATATTTATAAAGCTGGACGGAATACTTGTTGCCGC
bip  CCCCGCGGCTGTATTCCTTGCCACTCAATTTATTAAATTAGTTAAATATTTATGAAGCTG-CAGAAC---TTGTTGCCGC
mir  CCCTGCGGCTGGATTTCTTGCCACTCAATTTATTAAATTTATTAAATATTTATGAAGTTTGCCAA-C---ACATTTGCTC
pse  CCCTGCGGCTGGATTTCTTGCCACTCAATTTATTAAATTTATTAAATATTTATGAAGTTTGCCAA-C---ACATTTGCTC
per  CCCTGCGGCTGGATTTCTTGCCACTCAATTTATTAAATTTATTAAATATTTATGAAGTTTGCCAA-C---ACATTTGCTC
wil  N/A
gri  N/A
vir  N/A
moj  N/A
```

Supplementary Fig. 6

Supplementary Fig. 6. The conservation of *dsx-1* CRM sequence correlates with the presence of sex combs.

DNA sequence near the center of Hox ChIP peak 2 of the *dsx-1* CRM is aligned across selected *Drosophila* species. The grey bar highlights the region matches the region denoted by the vertical grey bar in Fig. 8B, and is the region of the CRM that when deleted, alters the *dsx-1* reporter gene expression. This region is highly AT-rich and contains many potential homeodomain binding sites, several of which fit the Scr-Dll consensus binding site. The presence or absence of sex comb teeth on male T1 legs is shown, and is based on [5,6].

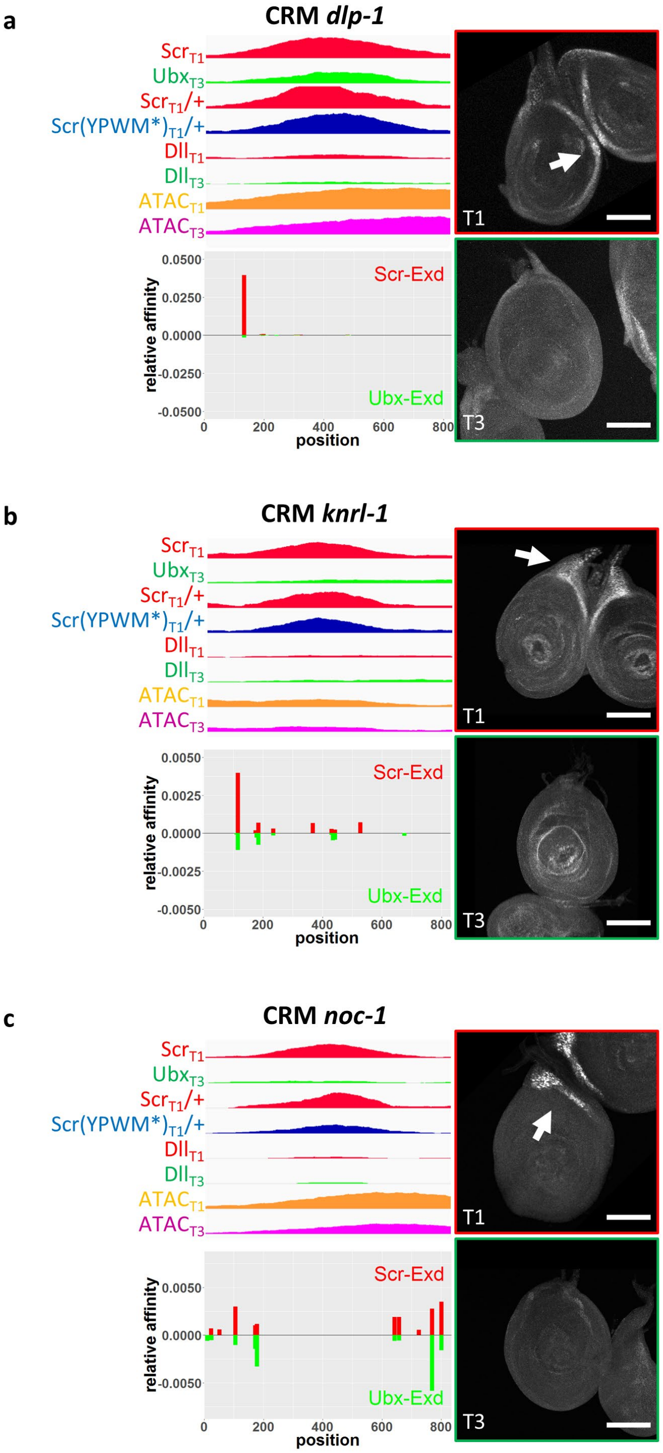

Supplementary Fig. 7

Supplementary Fig. 7. Examples of Exd-independent Scr_{T1}>Ubx_{T3} CRMs without Dll co-occupancy that drive T1>T3 expression

a. CRM *dlp-1*. **b.** CRM *knrl-1*. **c.** CRM *noc-1*. Antibody staining results of T1 and T3 leg discs are shown, and the T1 specific expression patterns are indicated by arrows. The genome browser tracks for the Scr_{T1}, Ubx_{T3}, Scr_{T1}/+, Scr(YPWM*)$_{T1}$/+, Dll_{T1} and Dll_{T3} ChIP-seq signals, as well as $ATAC_{T1}$ and $ATAC_{T3}$ signals are shown for the genomic fragment used to generate the reporters (top left). The *NRLB* relative affinity tracks for Scr-Exd and Ubx-Exd heterodimers, aligned with the genome browser tracks, are shown at the bottom left. Note the absence of significant Hox-Exd binding motifs near Scr ChIP-seq peak center. Scale bar: 100 µm.

The second *Scr* exon (the first coding exon plus part of 5' UTR)

ACTCCAATCCACGACAACGACCCCTGGCTTTAAAATGGATCCCGACTGTTTTGCGA
TGTCCTCGTACCAGT**TCGTCAACTCGCTGGCCT**CCTGTTATCCGCAGC**AGATGAA**
TCCGCAGCAGAATCACCCGGGAGCGGGAAATTCGAGTGCAGGCGGCAGCGGTG
GAGGGGCGGGCGGTAGCGGGGGCGTGGTGCCCAGCGGCGGAACGAACGGCGG
TCAGGGCAGTGCGGGGGCGGCCACTCCGGGGGCGAACGACTACTTCCCAGCCG
CGGCGGCCTATACGCCTAACCTGTATCCAAACACACCGCAGGCCCACTACGCCAA
TCAGGCGGCCTACGGCGGACAGGGAAATCCGGATATGGTGGACTACACGCAGCT
ACAGCCGCAGCGACTGCTCCTGCAGCAGCAGCAGCAACAGCAACAGCAGCAACA
TGCCCATGCAGCGGCAGCAGTTGCTGCCCAGCAGCAGCAGCAACTCGCCCAGCA
GCAACACCCGCAACAGCAGCAACAGCAGCAGCAGGCGAACATCAGTTGCAAGTAC
GCCAACGATCCAGTGACGCCGGGCGGAAGTGGGGGCGGGGGTGTTTCCGGTAG
CAACAACAATAACAATAGCGCCAACAGCAACAACAACAACAGCCAGTCGCTAGCCA
GTCCACAGGATCTGTCCACCAGGGACATATCGCCCAAACTGTCGCCCAGCTCAGT
GGTAGAGAGTGTGGCCCGATCGCTGAACAAGGGCGTCCTGGGCGGAAGCTTGGC
GGCCGCCGCTGCCGCCGCCGGCTTGAACAACAACCACAGCGGATCGGGAGTGTC
CGGAGGACCTGGAAACGTCAATGTTCCCATGCACAGTCCGGGGGGCGGGGATTC
GGACTCCGAGAGCGATAGCGGCAATGAGGCGGGCAGTAGCCAGAACAGCGGCAA
TGGAAAGAAGAACCCGCCGCAGATATATCCGTGGATGAAGCGAGTACATCTCGGG
ACGA

ATG start codon of *Scr*
YPWM motif encoding sequence
***Scr*-TALEN-L and *Scr*-TALEN-R target sites**
TALEN target spacer
47 bp deleted in the *Scr*$^{C8\text{-}1}$ allele

Scr-DDD TALEN Sequence (pCS2TAL3-Scr-DDD)
Primer Sequences
ATG-Start Codon
Flag Tag
RVD Sequence
Unique Restriction Enzyme Sites
FokI Domain-DDD
SV40 UTR

CGCCATTCTGCCTGGGGACGTCGGAGCAAGCTTGATTTAGGTGACACTATAGAAT
ACAAGCTACTTGTTCTTTTTGCAGGATCTGCCACCATGGCTCCAAAGAAGAAGCGT
AAGGTAGACTACAAAGACCATGACGGTGATTATAAAGATCATGACATCGATTACAA
GGATGACGATGACAAGGGTACCGTGGATCTACGCACGCTCGGCTACAGTCAGCAG
CAGCAAGAGAAGATCAAACCGAAGGTGCGTTCGACAGTGGCGCAGCACCACGAG
GCACTGGTGGGCCATGGGTTTACACACGCGCACATCGTTGCGCTCAGCCAACACC
CGGCAGCGTTAGGGACCGTCGCTGTCACGTATCAGCACATAATCACGGCGTTGCC
AGAGGCGACACACGAAGACATCGTTGGCGTCGGCAAACAGTGGTCCGGCGCACG

CGCCCTGGAGGCCTTGCTCACGGATGCGGGGGAGTTGAGAGGTCCGCCGTTACA
GTTGGACACAGGCCAACTTGTGAAGATTGCAAAACGTGGCGGCGTGACCGCAATG
GAGGCAGTGCATGCATCGCGCAATGCACTGACGGGTGCCCCCCT
GAACCTGACCCCGGACCAAGTGGTGGCTATCGCCAGCCACGATGGCGGCAAGCA
AGCGCTCGAAACGGTGCAGCGGCTGTTGCCGGTGCTGTGCCAGGACCATGGCCT
GACCCCGGACCAAGTGGTGGCTATCGCCAGCAACAATGGCGGCAAGCAAGCGCT
CGAAACGGTGCAGCGGCTGTTGCCGGTGCTGTGCCAGGACCATGGCCTGACCCC
GGACCAAGTGGTGGCTATCGCCAGCAACGGTGGCGGCAAGCAAGCGCTCGAAAC
GGTGCAGCGGCTGTTGCCGGTGCTGTGCCAGGACCATGGCCTGACTCCGGACCA
AGTGGTGGCTATCGCCAGCCACGATGGCGGCAAGCAAGCGCTCGAAACGGTGCA
GCGGCTGTTGCCGGTGCTGTGCCAGGACCATGGCCTGACCCCGGACCAAGTGGT
GGCTATCGCCAGCAACATTGGCGGCAAGCAAGCGCTCGAAACGGTGCAGCGGCT
GTTGCCGGTGCTGTGCCAGGACCATGGCCTGACCCCGGACCAAGTGGTGGCTAT
CGCCAGCAACATTGGTGGAAAACAGGCCCTTGAAACGGTGCAGCGGCTGTTGCC
GGTGCTGTGCCAGGACCATGGCCTGACTCCGGACCAAGTGGTGGCTATCGCCAG
CCACGATGGCGGCAAGCAAGCGCTCGAAACGGTGCAGCGGCTGTTGCCGGTGCT
GTGCCAGGACCATGGCCTGACCCCGGACCAAGTGGTGGCTATCGCCAGCAACGG
TGGCGGCAAGCAAGCGCTCGAAACGGTGCAGCGGCTGTTGCCGGTGCTGTGCCA
GGACCATGGCCTGACTCCGGACCAAGTGGTGGCTATCGCCAGCCACGATGGCGG
CAAGCAAGCGCTCGAAACGGTGCAGCGGCTGTTGCCGGTGCTGTGCCAGGACCA
TGGCCTGACCCCGGACCAAGTGGTGGCTATCGCCAGCAACAATGGCGGCAAGCA
AGCGCTCGAAACGGTGCAGCGGCTGTTGCCGGTGCTGTGCCAGGACCATGGCCT
GACCCCGGACCAAGTGGTGGCTATCGCCAGCCACGATGGCGGCAAGCAAGCGCT
CGAAACGGTGCAGCGGCTGTTGCCGGTGCTGTGCCAGGACCATGGCCTGACCCC
GGACCAAGTGGTGGCTATCGCCAGCAACGGTGGCGGCAAGCAAGCGCTCGAAAC
GGTGCAGCGGCTGTTGCCGGTGCTGTGCCAGGACCATGGCCTGACCCCGGACCA
AGTGGTGGCTATCGCCAGCAACAATGGCGGCAAGCAAGCGCTCGAAACGGTGCA
GCGGCTGTTGCCGGTGCTGTGCCAGGACCATGGCCTGACCCCGGACCAAGTGGT
GGCTATCGCCAGCAACAATGGCGGCAAGCAAGCGCTCGAAACGGTGCAGCGGCT
GTTGCCGGTGCTGTGCCAGGACCATGGCCTGACTCCGGACCAAGTGGTGGCTATC
GCCAGCCACGATGGCGGCAAGCAAGCGCTCGAAACGGTGCAGCGGCTGTTGCCG
GTGCTGTGCCAGGACCATGGCCTGACTCCGGACCAAGTGGTGGCTATCGCCAGC
CACGATGGCGGCAAGCAAGCGCTCGAAACGGTGCAGCGGCTGTTGCCGGTGCTG
TGCCAGGACCATGGCCTGACCCCGGACCAAGTGGTGGCTATCGCCAGCAACGGT
GGCGGCAAGCAAGCGCTCGAAAGCATTGTGGCCCAGCTGAGCCGGCCTGATCCG
GCGTTGGCCGCGTTGAC
CAACGACCACCTCGTCGCCTTGGCCTGCCTCGGCGGACGTCCTGCCATGGATGCA
GTGAAAAAGGGATTGCCGCACGCGCCGGAATTGATCAGAAGAGTCAATCGCCGTA
TTGGCGAACGCACGTCCCATCGCGTTGCCGGATCCCAACTAGTCAAAAGTGAACT
GGAGGAGAAGAAATCTGAACTTCGTCATAAATTGAAATATGTGCCTCATGAATATAT
TGAATTAATTGAAATTGCCAGAAATTCCACTCAGGATAGAATTCTTGAAATGAAGGT
AATGGAATTTTTTATGAAAGTTTATGGATATAGAGGTAAACATTTGGGTGGATCAAG
GAAACCGGACGGAGCAATTTATACTGTCGGATCTCCTATTGATTACGGTGTGATCG
TGGATACTAAAGCTTATAGCGGAGGTTATAATCTGCCAATTGGCCAAGCAGATGAA
ATGCAAGACTATGTCGAAGAAAATCAAACACGAGACAAACATATCAACCCTAATGA
ATGGTGGAAAGTCTATCCATCTTCTGTAACGGAATTTAAGTTTTTATTTGTGAGTGG

TCACTTTAAAGGAAACTACAAAGCTCAGCTTACACGATTAAATCATATCACTAATTG
TAATGGAGCTGTTCTTAGTGTAGAAGAGCTTTTAATTGGTGGAGAAATGATTAAAGC
CGGCACATTAACCTTAGAGGAAGTGCGCCGGAAATTTAATAACGGCGAGATAAACT
TTTAATCTAGAACTATAGTGAGTCGTATTACGTAGATCCAGACATGATAAGATACAT
TGATGAGTTTGGACAAACCACAACTAGAATGCAGTGAAAAAAATGCTTTATTTGTGA
AATTTGTGATGCTATTGCTTTATTTGTAACCATTATAAGCTGCAATAAACAAGTTAAC
AACAACAATTGCATTCATTTTATGTTTCAGGTTCAGGGGGAGGTGTGGGAGGTTTT
TTAATTCGCGGCCGCGGCGCCAATGCATTGGGCCCGGTACGTACCCAGCTTTTGT
TCCCTTTAGTGAGGGTTAATTGCGCGCTTGGCGTAATCATGGTCATAGCTGTTTCC
TGTGTGAAATTGTTATCCGCTCACAATTCCACACAACATACGAGCCGGAAGCATAA
AGTGTAAAGCCTGGGGTGCCTAATGAGTGAGCTAACTCACATTAATTGCGTTGCGC
TCACTGCCCGCTTTCCAGTCGGGAAACCTGTCGTGCCAGCTGCATTAATGAATCG
GCCAACGCGCGGGGAGAGGCGGTTTGCGTATTGGGCGCTCTTCCGCTTCCTCGC
TCACTGACTCGCTGCGCTCGGTCGTTCGGCTGCGGCGAGCGGTATCAGCTCACTC
AAAGGCGGTAATACGGTTATCCACAGAATCAGGGGATAACGCAGGAAAGAACATG
TGAGCAAAAGGCCAGCAAAAGGCCAGGAACCGTAAAAAGGCCGCGTTGCTGGCG
TTTTTCCATAGGCTCCGCCCCCCTGACGAGCATCACAAAAATCGACGCTCAAGTCA
GAGGTGGCGAAACCCGACAGGACTATAAAGATACCAGGCGTTTCCCCCTGGAAGC
TCCCTCGTGCGCTCTCCTGTTCCGACCCTGCCGCTTACCGGATACCTGTCCGCCT
TTCTCCCTTCGGGAAGCGTGGCGCTTTCTCATAGCTCACGCTGTAGGTATCTCAGT
TCGGTGTAGGTCGTTCGCTCCAAGCTGGGCTGTGTGCACGAACCCCCCGTTCAGC
CCGACCGCTGCGCCTTATCCGGTAACTATCGTCTTGAGTCCAACCCGGTAAGACA
CGACTTATCGCCACTGGCAGCAGCCACTGGTAACAGGATTAGCAGAGCGAGGTAT
GTAGGCGGTGCTACAGAGTTCTTGAAGTGGTGGCCTAACTACGGCTACACTAGAA
GGACAGTATTTGGTATCTGCGCTCTGCTGAAGCCAGTTACCTTCGGAAAAAGAGTT
GGTAGCTCTTGATCCGGCAAACAAACCACCGCTGGTAGCGGTGGTTTTTTTGTTTG
CAAGCAGCAGATTACGCGCAGAAAAAAAGGATCTCAAGAAGATCCTTTGATCTTTT
CTACGGGGTCTGACGCTCAGTGGAACGAAAACTCACGTTAAGGGATTTTGGTCAT
GAGATTATCAAAAAGGATCTTCACCTAGATCCTTTTAAATTAAAAATGAAGTTTTAAA
TCAATCTAAAGTATATATGAGTAAACTTGGTCTGACAGTTACCAATGCTTAATCAGT
GAGGCACCTATCTCAGCGATCTGTCTATTTCGTTCATCCATAGTTGCCTGACTCCC
CGTCGTGTAGATAACTACGATACGGGAGGGCTTACCATCTGGCCCCAGTGCTGCA
ATGATACCGCGAGACCCACGCTCACCGGCTCCAGATTTATCAGCAATAAACCAGC
CAGCCGGAAGGGCCGAGCGCAGAAGTGGTCCTGCAACTTTATCCGCCTCCATCCA
GTCTATTAATTGTTGCCGGGAAGCTAGAGTAAGTAGTTCGCCAGTTAATAGTTTGC
GCAACGTTGTTGCCATTGCTACAGGCATCGTGGTGTCACGCTCGTCGTTTGGTATG
GCTTCATTCAGCTCCGGTTCCCAACGATCAAGGCGAGTTACATGATCCCCCATGTT
GTGCAAAAAAGCGGTTAGCTCCTTCGGTCCTCCGATCGTTGTCAGAAGTAAGTTGG
CCGCAGTGTTATCACTCATGGTTATGGCAGCACTGCATAATTCTCTTACTGTCATGC
CATCCGTAAGATGCTTTTCTGTGACTGGTGAGTACTCAACCAAGTCATTCTGAGAAT
AGTGTATGCGGCGACCGAGTTGCTCTTGCCCGGCGTCAATACGGGATAATACCGC
GCCACATAGCAGAACTTTAAAAGTGCTCATCATTGGAAAACGTTCTTCGGGGCGAA
AACTCTCAAGGATCTTACCGCTGTTGAGATCCAGTTCGATGTAACCCACTCGTGCA
CCCAACTGATCTTCAGCATCTTTTACTTTCACCAGCGTTTCTGGGTGAGCAAAAACA
GGAAGGCAAAATGCCGCAAAAAAGGGAATAAGGGCGACACGGAAATGTTGAATAC
TCATACTCTTCCTTTTTCAATATTATTGAAGCATTTATCAGGGTTATTGTCTCATGAG

CGGATACATATTTGAATGTATTTAGAAAAATAAACAAATAGGGGTTCCGCGCACATT
TCCCCGAAAAGTGCCACCTAAATTGTAAGCGTTAATATTTTGTTAAAATTCGCGTTA
AATTTTTGTTAAATCAGCTCATTTTTTAACCAATAGGCCGAAATCGGCAAAATCCCT
TATAAATCAAAAGAATAGACCGAGATAGGGTTGAGTGTTGTTCCAGTTTGGAACAA
GAGTCCACTATTAAAGAACGTGGACTCCAACGTCAAAGGGCGAAAAACCGTCTATC
AGGGCGATGGCCCACTACGTGAACCATCACCCTAATCAAGTTTTTTGGGGTCGAG
GTGCCGTAAAGCACTAAATCGGAACCCTAAAGGGAGCCCCCGATTTAGAGCTTGA
CGGGGAAAGCCGGCGAACGTGGCGAGAAAGGAAGGGAAGAAAGCGAAAGGAGC
GGGCGCTAGGGCGCTGGCAAGTGTAGCGGTCACGCTGCGCGTAACCACCACACC
CGCCGCGCTTAATGCGCCGCTACAGGGCGCGTCCCATTCGCCATTCAGGCTGCG
CAACTGTTGGGAAGGGCGATCGGTGCGGGCCTCTTCGCTATTACGCCAGTCGACC
ATAGCCAATTCAATATGGCGTATATGGACTCATGCCAATTCAATATGGTGGATCTG
GACCTGTGCCAATTCAATATGGCGTATATGGACTCGTGCCAATTCAATATGGTGGA
TCTGGACCCCAGCCAATTCAATATGGCGGACTTGGCACCATGCCAATTCAATATGG
CGGACTTGGCACTGTGCCAACTGGGGAGGGGTCTACTTGGCACGGTGCCAAGTTT
GAGGAGGGGTCTTGGCCCTGTGCCAAGTCCGCCATATTGAATTGGCATGGTGCCA
ATAATGGCGGCCATATTGGCTATATGCCAGGATCAATATATAGGCAATATCCAATAT
GGCCCTATGCCAATATGGCTATTGGCCAGGTTCAATACTATGTATTGGCCCTATGC
CATATAGTATTCCATATATGGGTTTTCCTATTGACGTAGATAGCCCCTCCCAATGGG
CGGTCCCATATACCATATATGGGGCTTCCTAATACCGCCCATAGCCACTCCCCCAT
TGACGTCAATGGTCTCTATATATGGTCTTTCCTATTGACGTCATATGGGCGGTCCTA
TTGACGTATATGGCGCCTCCCCCATTGACGTCAATTACGGTAAATGGCCCGCCTG
GCTCAATGCCCATTGACGTCAATAGGACCACCCACCATTGACGTCAATGGGATGG
CTCATTGCCCATTCATATCCGTTCTCACGCCCCCTATTGACGTCAATGACGGTAAAT
GGCCCACTTGGCAGTACATCAATATCTATTAATAGTAACTTGGCAAGTACATTACTA
TTGGAAGGACGCCAGGGTACATTGGCAGTACTCCCATTGACGTCAATGGCGGTAA
ATGGCCCGCGATGGCTGCCAAGTACATCCCCATTGACGTCAATGGGGAGGGGCA
ATGACGCAAATGGGCGTTCCATTGACGTAAATGGGCGGTAGGCGTGCCTAATGGG
AGGTCTATATAAGCAATGCTCGTTTAGGGAAC

Scr-RRR TALEN Sequence (pCS2TAL3-Scr-RRR)
Primer Sequences
ATG-Start Codon
HA TAG
RVD Sequence
Unique Restriction Enzyme Sites
FokI Domain-RRR
SV40 UTR

CGCCATTCTGCCTGGGGACGTCGGAGCAAGCTTGATTTAGGTGACACTATAGAAT
ACAAGCTACTTGTTCTTTTTGCAGGATCTGCCACCATGGCTCCAAAGAAGAAGCGT
AAGGTATACCCATACGATGTTCCTGACTATGCGGGCTATCCCTATGACGTCCCGGA
CTATGCAGGATCGTATCCATATGACGTTCCAGATTACGCTGCTCATGGTACCGTGG
ATCTACGCACGCTCGGCTACAGTCAGCAGCAGCAAGAGAAGATCAAACCGAAGGT
GCGTTCGACAGTGGCGCAGCACCACGAGGCACTGGTGGGCCATGGGTTTACACA
CGCGCACATCGTTGCGCTCAGCCAACACCCGGCAGCGTTAGGGACCGTCGCTGT

CACGTATCAGCACATAATCACGGCGTTGCCAGAGGCGACACACGAAGACATCGTT
GGCGTCGGCAAACAGTGGTCCGGCGCACGCGCCCTGGAGGCCTTGCTCACGGAT
GCGGGGGAGTTGAGAGGTCCGCCGTTACAGTTGGACACAGGCCAACTTGTGAAG
ATTGCAAAACGTGGCGGCGTGACCGCAATGGAGGCAGTGCATGCATCGCGCAAT
GCACTGACGGGTGCCCCCCT
GAACCTGACCCCGGACCAAGTGGTGGCTATCGCCAGCCACGATGGCGGCAAGCA
AGCGCTCGAAACGGTGCAGCGGCTGTTGCCGGTGCTGTGCCAGGACCATGGCCT
GACCCCGGACCAAGTGGTGGCTATCGCCAGCAACGGTGGCGGCAAGCAAGCGCT
CGAAACGGTGCAGCGGCTGTTGCCGGTGCTGTGCCAGGACCATGGCCTGACCCC
GGACCAAGTGGTGGCTATCGCCAGCAACAATGGCGGCAAGCAAGCGCTCGAAAC
GGTGCAGCGGCTGTTGCCGGTGCTGTGCCAGGACCATGGCCTGACTCCGGACCA
AGTGGTGGCTATCGCCAGCCACGATGGCGGCAAGCAAGCGCTCGAAACGGTGCA
GCGGCTGTTGCCGGTGCTGTGCCAGGACCATGGCCTGACCCCGGACCAAGTGGT
GGCTATCGCCAGCAACGGTGGCGGCAAGCAAGCGCTCGAAACGGTGCAGCGGCT
GTTGCCGGTGCTGTGCCAGGACCATGGCCTGACCCCGGACCAAGTGGTGGCTAT
CGCCAGCAACAATGGTGGAAAACAGGCCCTTGAAACGGTGCAGCGGCTGTTGCC
GGTGCTGTGCCAGGACCATGGCCTGACTCCGGACCAAGTGGTGGCTATCGCCAG
CCACGATGGCGGCAAGCAAGCGCTCGAAACGGTGCAGCGGCTGTTGCCGGTGCT
GTGCCAGGACCATGGCCTGACCCCGGACCAAGTGGTGGCTATCGCCAGCAACAA
TGGCGGCAAGCAAGCGCTCGAAACGGTGCAGCGGCTGTTGCCGGTGCTGTGCCA
GGACCATGGCCTGACCCCGGACCAAGTGGTGGCTATCGCCAGCAACAATGGCGG
CAAGCAAGCGCTCGAAACGGTGCAGCGGCTGTTGCCGGTGCTGTGCCAGGACCA
TGGCCTGACCCCGGACCAAGTGGTGGCTATCGCCAGCAACATTGGCGGCAAGCA
AGCGCTCGAAACGGTGCAGCGGCTGTTGCCGGTGCTGTGCCAGGACCATGGCCT
GACCCCGGACCAAGTGGTGGCTATCGCCAGCAACGGTGGCGGCAAGCAAGCGCT
CGAAACGGTGCAGCGGCTGTTGCCGGTGCTGTGCCAGGACCATGGCCTGACCCC
GGACCAAGTGGTGGCTATCGCCAGCAACGGTGGCGGCAAGCAAGCGCTCGAAAC
GGTGCAGCGGCTGTTGCCGGTGCTGTGCCAGGACCATGGCCTGACTCCGGACCA
AGTGGTGGCTATCGCCAGCCACGATGGCGGCAAGCAAGCGCTCGAAACGGTGCA
GCGGCTGTTGCCGGTGCTGTGCCAGGACCATGGCCTGACCCCGGACCAAGTGGT
GGCTATCGCCAGCAACATTGGCGGCAAGCAAGCGCTCGAAACGGTGCAGCGGCT
GTTGCCGGTGCTGTGCCAGGACCATGGCCTGACCCCGGACCAAGTGGTGGCTAT
CGCCAGCAACGGTGGCGGCAAGCAAGCGCTCGAAACGGTGCAGCGGCTGTTGCC
GGTGCTGTGCCAGGACCATGGCCTGACTCCGGACCAAGTGGTGGCTATCGCCAG
CCACGATGGCGGCAAGCAAGCGCTCGAAACGGTGCAGCGGCTGTTGCCGGTGCT
GTGCCAGGACCATGGCCTGACCCCGGACCAAGTGGTGGCTATCGCCAGCAACGG
TGGCGGCAAGCAAGCGCTCGAAAGCATTGTGGCCCAGCTGAGCCGGCCTGATCC
GGCGTTGGCCGCGTTGAC
CAACGACCACCTCGTCGCCTTGGCCTGCCTCGGCGGACGTCCTGCCATGGATGCA
GTGAAAAAGGGATTGCCGCACGCGCCGGAATTGATCAGAAGAGTCAATCGCCGTA
TTGGCGAACGCACGTCCCATCGCGTTGCCGGATCCCAACTAGTCAAAAGTGAACT
GGAGGAGAAGAAATCTGAACTTCGTCATAAATTGAAATATGTGCCTCATGAATATAT
TGAATTAATTGAAATTGCCAGAAATTCCACTCAGGATAGAATTCTTGAAATGAAGGT
AATGGAATTTTTTATGAAAGTTTATGGATATAGAGGTAAACATTTGGGTGGATCAAG
GAAACCGGACGGAGCAATTTATACTGTCGGATCTCCTATTGATTACGGTGTGATCG
TGGATACTAAAGCTTATAGCGGAGGTTATAATCTGCCAATTGGCCAAGCACGTGAA

ATGCAACGATATGTCGAAGAAAATCAAACACGAAACAAACATATCAACCCTAATGAA
TGGTGGAAAGTCTATCCATCTTCTGTAACGGAATTTAAGTTTTTATTTGTGAGTGGT
CACTTTAAAGGAAACTACAAAGCTCAGCTTACACGATTAAATAGAATCACTAATTGT
AATGGAGCTGTTCTTAGTGTAGAAGAGCTTTTAATTGGTGGAGAAATGATTAAAGC
CGGCACATTAACCTTAGAGGAAGTGCGCCGGAAATTTAATAACGGCGAGATAAACT
TTTAATCTAGAACTATAGTGAGTCGTATTACGTAGATCCAGACATGATAAGATACAT
TGATGAGTTTGGACAAACCACAACTAGAATGCAGTGAAAAAAATGCTTTATTTGTGA
AATTTGTGATGCTATTGCTTTATTTGTAACCATTATAAGCTGCAATAAACAAGTTAAC
AACAACAATTGCATTCATTTTATGTTTCAGGTTCAGGGGGAGGTGTGGGAGGTTTT
TTAATTCGCGGCCGCGGCGCCAATGCATTGGGCCCGGTACGTACCCAGCTTTTGT
TCCCTTTAGTGAGGGTTAATTGCGCGCTTGGCGTAATCATGGTCATAGCTGTTTCC
TGTGTGAAATTGTTATCCGCTCACAATTCCACACAACATACGAGCCGGAAGCATAA
AGTGTAAAGCCTGGGGTGCCTAATGAGTGAGCTAACTCACATTAATTGCGTTGCGC
TCACTGCCCGCTTTCCAGTCGGGAAACCTGTCGTGCCAGCTGCATTAATGAATCG
GCCAACGCGCGGGGAGAGGCGGTTTGCGTATTGGGCGCTCTTCCGCTTCCTCGC
TCACTGACTCGCTGCGCTCGGTCGTTCGGCTGCGGCGAGCGGTATCAGCTCACTC
AAAGGCGGTAATACGGTTATCCACAGAATCAGGGGATAACGCAGGAAAGAACATG
TGAGCAAAAGGCCAGCAAAAGGCCAGGAACCGTAAAAAGGCCGCGTTGCTGGCG
TTTTTCCATAGGCTCCGCCCCCCTGACGAGCATCACAAAAATCGACGCTCAAGTCA
GAGGTGGCGAAACCCGACAGGACTATAAAGATACCAGGCGTTTCCCCCTGGAAGC
TCCCTCGTGCGCTCTCCTGTTCCGACCCTGCCGCTTACCGGATACCTGTCCGCCT
TTCTCCCTTCGGGAAGCGTGGCGCTTTCTCATAGCTCACGCTGTAGGTATCTCAGT
TCGGTGTAGGTCGTTCGCTCCAAGCTGGGCTGTGTGCACGAACCCCCCGTTCAGC
CCGACCGCTGCGCCTTATCCGGTAACTATCGTCTTGAGTCCAACCCGGTAAGACA
CGACTTATCGCCACTGGCAGCAGCCACTGGTAACAGGATTAGCAGAGCGAGGTAT
GTAGGCGGTGCTACAGAGTTCTTGAAGTGGTGGCCTAACTACGGCTACACTAGAA
GGACAGTATTTGGTATCTGCGCTCTGCTGAAGCCAGTTACCTTCGGAAAAAGAGTT
GGTAGCTCTTGATCCGGCAAACAAACCACCGCTGGTAGCGGTGGTTTTTTTGTTTG
CAAGCAGCAGATTACGCGCAGAAAAAAAGGATCTCAAGAAGATCCTTTGATCTTTT
CTACGGGGTCTGACGCTCAGTGGAACGAAAACTCACGTTAAGGGATTTTGGTCAT
GAGATTATCAAAAAGGATCTTCACCTAGATCCTTTTAAATTAAAAATGAAGTTTTAAA
TCAATCTAAAGTATATATGAGTAAACTTGGTCTGACAGTTACCAATGCTTAATCAGT
GAGGCACCTATCTCAGCGATCTGTCTATTTCGTTCATCCATAGTTGCCTGACTCCC
CGTCGTGTAGATAACTACGATACGGGAGGGCTTACCATCTGGCCCCAGTGCTGCA
ATGATACCGCGAGACCCACGCTCACCGGCTCCAGATTTATCAGCAATAAACCAGC
CAGCCGGAAGGGCCGAGCGCAGAAGTGGTCCTGCAACTTTATCCGCCTCCATCCA
GTCTATTAATTGTTGCCGGGAAGCTAGAGTAAGTAGTTCGCCAGTTAATAGTTTGC
GCAACGTTGTTGCCATTGCTACAGGCATCGTGGTGTCACGCTCGTCGTTTGGTATG
GCTTCATTCAGCTCCGGTTCCCAACGATCAAGGCGAGTTACATGATCCCCCATGTT
GTGCAAAAAAGCGGTTAGCTCCTTCGGTCCTCCGATCGTTGTCAGAAGTAAGTTGG
CCGCAGTGTTATCACTCATGGTTATGGCAGCACTGCATAATTCTCTTACTGTCATGC
CATCCGTAAGATGCTTTTCTGTGACTGGTGAGTACTCAACCAAGTCATTCTGAGAAT
AGTGTATGCGGCGACCGAGTTGCTCTTGCCCGGCGTCAATACGGGATAATACCGC
GCCACATAGCAGAACTTTAAAAGTGCTCATCATTGGAAAACGTTCTTCGGGGCGAA
AACTCTCAAGGATCTTACCGCTGTTGAGATCCAGTTCGATGTAACCCACTCGTGCA
CCCAACTGATCTTCAGCATCTTTTACTTTCACCAGCGTTTCTGGGTGAGCAAAAACA

GGAAGGCAAAATGCCGCAAAAAAGGGAATAAGGGCGACACGGAAATGTTGAATAC
TCATACTCTTCCTTTTTCAATATTATTGAAGCATTTATCAGGGTTATTGTCTCATGAG
CGGATACATATTTGAATGTATTTAGAAAAATAAACAAATAGGGGTTCCGCGCACATT
TCCCCGAAAAGTGCCACCTAAATTGTAAGCGTTAATATTTTGTTAAAATTCGCGTTA
AATTTTTGTTAAATCAGCTCATTTTTTAACCAATAGGCCGAAATCGGCAAAATCCCT
TATAAATCAAAAGAATAGACCGAGATAGGGTTGAGTGTTGTTCCAGTTTGGAACAA
GAGTCCACTATTAAAGAACGTGGACTCCAACGTCAAAGGGCGAAAAACCGTCTATC
AGGGCGATGGCCCACTACGTGAACCATCACCCTAATCAAGTTTTTTGGGGTCGAG
GTGCCGTAAAGCACTAAATCGGAACCCTAAAGGGAGCCCCCGATTTAGAGCTTGA
CGGGGAAAGCCGGCGAACGTGGCGAGAAAGGAAGGGAAGAAAGCGAAAGGAGC
GGGCGCTAGGGCGCTGGCAAGTGTAGCGGTCACGCTGCGCGTAACCACCACACC
CGCCGCGCTTAATGCGCCGCTACAGGGCGCGTCCCATTCGCCATTCAGGCTGCG
CAACTGTTGGGAAGGGCGATCGGTGCGGGCCTCTTCGCTATTACGCCAGTCGACC
ATAGCCAATTCAATATGGCGTATATGGACTCATGCCAATTCAATATGGTGGATCTG
GACCTGTGCCAATTCAATATGGCGTATATGGACTCGTGCCAATTCAATATGGTGGA
TCTGGACCCCAGCCAATTCAATATGGCGGACTTGGCACCATGCCAATTCAATATGG
CGGACTTGGCACTGTGCCAACTGGGGAGGGGTCTACTTGGCACGGTGCCAAGTTT
GAGGAGGGGTCTTGGCCCTGTGCCAAGTCCGCCATATTGAATTGGCATGGTGCCA
ATAATGGCGGCCATATTGGCTATATGCCAGGATCAATATATAGGCAATATCCAATAT
GGCCCTATGCCAATATGGCTATTGGCCAGGTTCAATACTATGTATTGGCCCTATGC
CATATAGTATTCCATATATGGGTTTTCCTATTGACGTAGATAGCCCCTCCCAATGGG
CGGTCCCATATACCATATATGGGGCTTCCTAATACCGCCCATAGCCACTCCCCCAT
TGACGTCAATGGTCTCTATATATGGTCTTTCCTATTGACGTCATATGGGCGGTCCTA
TTGACGTATATGGCGCCTCCCCCATTGACGTCAATTACGGTAAATGGCCCGCCTG
GCTCAATGCCCATTGACGTCAATAGGACCACCCACCATTGACGTCAATGGGATGG
CTCATTGCCCATTCATATCCGTTCTCACGCCCCCTATTGACGTCAATGACGGTAAAT
GGCCCACTTGGCAGTACATCAATATCTATTAATAGTAACTTGGCAAGTACATTACTA
TTGGAAGGACGCCAGGGTACATTGGCAGTACTCCCATTGACGTCAATGGCGGTAA
ATGGCCCGCGATGGCTGCCAAGTACATCCCCATTGACGTCAATGGGGAGGGGCA
ATGACGCAAATGGGCGTTCCATTGACGTAAATGGGCGGTAGGCGTGCCTAATGGG
AGGTCTATATAAGCAATGCTCGTTTAGGGAAC

References

1. Pattatucci, A.M. & Kaufman, T.C. The homeotic gene Sex combs reduced of Drosophila melanogaster is differentially regulated in the embryonic and imaginal stages of development. *Genetics* **129**, 443-61 (1991).
2. Feng, S., Lu, S., Grueber, W.B. & Mann, R.S. Scarless engineering of the Drosophila genome near any site-specific integration site. *Genetics* **217**(2021).
3. Slattery, M. *et al.* Cofactor binding evokes latent differences in DNA binding specificity between Hox proteins. *Cell* **147**, 1270-82 (2011).
4. Tsubota, T., Saigo, K. & Kojima, T. Hox genes regulate the same character by different strategies in each segment. *Mechanisms of Development* **125**, 894-905 (2008).
5. Dobzhansky, T. DROSOPHILA MIRANDA, A NEW SPECIES. *Genetics* **20**, 377 (1935).
6. Kopp, A. Drosophila sex combs as a model of evolutionary innovations. *Evol Dev* **13**, 504-22 (2011).
