# Supplementary material for: Transcription factor paralogs orchestrate alternative gene regulatory networks by context-dependent cooperation with multiple cofactors
Source: Nat Commun. 2022 Jul 1;13:3808. doi: 10.1038/s41467-022-31501-2 (PMC9249852; doi:10.1038/s41467-022-31501-2)
Supplement: Supplementary file 3 — Description of additional Supplementary File [file 41467_2022_31501_MOESM3_ESM.pdf]

<u>Descriptions of additional supplementary information files</u>

1. Supplementary Data 1 – transcriptome details
2. Supplementary Data 2 – ChIP-seq details
3. Supplementary Data 3 -- primers
4. Supplementary Data 4 – reporter gene coordinates
