# Supplementary material for: Transcription factor paralogs orchestrate alternative gene regulatory networks by context-dependent cooperation with multiple cofactors
Source: Nat Commun. 2022 Jul 1;13:3808. doi: 10.1038/s41467-022-31501-2 (PMC9249852; doi:10.1038/s41467-022-31501-2)
Supplement: Supplementary file 8 — Reporting Summary [file 41467_2022_31501_MOESM8_ESM.pdf]

nature portfolio

Corresponding author(s): Richard S. Mann

Last updated by author(s): May 21, 2022

Reporting Summary

Nature Portfolio wishes to improve the reproducibility of the work that we publish. This form provides structure for consistency and transparency in reporting. For further information on Nature Portfolio policies, see our Editorial Policies and the Editorial Policy Checklist.

Statistics

For all statistical analyses, confirm that the following items are present in the figure legend, table legend, main text, or Methods section.

n/a	Confirmed	
☐	☒	The exact sample size (*n*) for each experimental group/condition, given as a discrete number and unit of measurement
☐	☒	A statement on whether measurements were taken from distinct samples or whether the same sample was measured repeatedly
☐	☒	The statistical test(s) used AND whether they are one- or two-sided<br>*Only common tests should be described solely by name; describe more complex techniques in the Methods section.*
☒	☐	A description of all covariates tested
☒	☐	A description of any assumptions or corrections, such as tests of normality and adjustment for multiple comparisons
☒	☐	A full description of the statistical parameters including central tendency (e.g. means) or other basic estimates (e.g. regression coefficient) AND variation (e.g. standard deviation) or associated estimates of uncertainty (e.g. confidence intervals)
☒	☐	For null hypothesis testing, the test statistic (e.g. *F*, *t*, *r*) with confidence intervals, effect sizes, degrees of freedom and *P* value noted<br>*Give P values as exact values whenever suitable.*
☒	☐	For Bayesian analysis, information on the choice of priors and Markov chain Monte Carlo settings
☒	☐	For hierarchical and complex designs, identification of the appropriate level for tests and full reporting of outcomes
☐	☒	Estimates of effect sizes (e.g. Cohen's *d*, Pearson's *r*), indicating how they were calculated

Our web collection on statistics for biologists contains articles on many of the points above.

Software and code

Policy information about availability of computer code

Data collection	No software was used for data collection
Data analysis	The following softwares were used in data analysis: bowtie (Galaxy Version 1.2.0 on usegalaxy.org, against genome build dm3), samtools (Galaxy Version 1.8+galaxy1 on usegalaxy.org), MACS2 (Galaxy Version 2.2.7.1+galaxy0 on usegalaxy.org), homer (V4.10), NRLBtools (V1.1), DiffBind (V2.12.0), Cutadapt (Galaxy Version 4.0+galaxy0 on usegalaxy.eu), RNA STAR (Galaxy Version 2.7.8a+galaxy0 on usegalaxy.eu against genome build dm6), featureCounts (Galaxy Version 2.0.1+galaxy2on usegalaxy.eu), DEseq2(V1.24.0)

For manuscripts utilizing custom algorithms or software that are central to the research but not yet described in published literature, software must be made available to editors and reviewers. We strongly encourage code deposition in a community repository (e.g. GitHub). See the Nature Portfolio guidelines for submitting code & software for further information.

Data

Policy information about availability of data

All manuscripts must include a data availability statement. This statement should provide the following information, where applicable:

- Accession codes, unique identifiers, or web links for publicly available datasets
- A description of any restrictions on data availability
- For clinical datasets or third party data, please ensure that the statement adheres to our policy

All next generation sequencing data have been uploaded to GEO, and can be accessed at GSE184454

Field-specific reporting

Please select the one below that is the best fit for your research. If you are not sure, read the appropriate sections before making your selection.

☒ Life sciences ☐ Behavioural & social sciences ☐ Ecological, evolutionary & environmental sciences

For a reference copy of the document with all sections, see nature.com/documents/nr-reporting-summary-flat.pdf

Life sciences study design

All studies must disclose on these points even when the disclosure is negative.

Sample size	The sample sizes (for example in Fig 3d) were determined empirically when applicable.
Data exclusions	No data were excluded
Replication	ChIP-seq and ATAC-seq experiments had at least 2 biological replicates, and RNA-seq experiments had at least 3. All biological replicates agreed with one another
Randomization	No experiment in this study required randomization.
Blinding	Researchers who analyzed the results were not blinded.

Reporting for specific materials, systems and methods

We require information from authors about some types of materials, experimental systems and methods used in many studies. Here, indicate whether each material, system or method listed is relevant to your study. If you are not sure if a list item applies to your research, read the appropriate section before selecting a response.

Materials & experimental systems

n/a	Involved in the study	
☐	☒	Antibodies
☒	☐	Eukaryotic cell lines
☒	☐	Palaeontology and archaeology
☐	☒	Animals and other organisms
☒	☐	Human research participants
☒	☐	Clinical data
☒	☐	Dual use research of concern

Methods

n/a	Involved in the study	
☐	☒	ChIP-seq
☒	☐	Flow cytometry
☒	☐	MRI-based neuroimaging

Antibodies

Antibodies used	mouse anti-FLAG M2 antibody (Sigma F1804), Guinea pig anti-Dll antibody (Mann lab), guinea pig anti-Scr (Mann lab), monoclonal mouse anti-Ubx (FP3.38, ascites), polyclonal rabbit anti-β-Gal (MP Biomedicals, cat #559762, lot #06825), polyclonal guinea pig anti-Hth (Mann lab), polyclonal goat-anti Dll (Santa Cruz sc-15858), polyclonal rabbit anti-CrebA (Andrew et al., 1997), chicken anti-GFP (abcam ab13970), mouse anti-T7 antibody (Millipore Sigma 69522), HRP conjugated anti-T7 antibody (Millipore Sigma 69048)
Validation	All antibodies used were either commercially available or previously published. Validation of commercial antibodies can be found at manufacturer's websites. Validation of mouse anti-Ubx is available at DSHB. Guinea pig anti-Dll antibody (ref: PMID: 18194655), guinea pig anti-Scr (ref: PMID: 20634319), and guinea pig anti-Hth (ref: PMID: 10398683) were validated in previous studies. Validation of the No new antibody was generated in this study. For all antibodies used in staining experiments, the results fit the expected patterns of the corresponding antigens.

Animals and other organisms

Policy information about studies involving animals; ARRIVE guidelines recommended for reporting animal research

Laboratory animals	Drosophila melanogaster. Various strains were used as specified in the manuscript. Animals of mixed sexes were used unless specified. Age (developmental stage) of animals were specified in manuscript.
Wild animals	No wild animals were used in this study
Field-collected samples	No field collected samples were used in this study
Ethics oversight	This study did not require ethical approval

Note that full information on the approval of the study protocol must also be provided in the manuscript.

ChIP-seq

Data deposition

- [x] Confirm that both raw and final processed data have been deposited in a public database such as GEO.
- [x] Confirm that you have deposited or provided access to graph files (e.g. BED files) for the called peaks.

Data access links<br>*May remain private before publication.*	https://www.ncbi.nlm.nih.gov/geo/query/acc.cgi?acc=GSE184454 token:eboxasuwlpwlvqx
Files in database submission	raw fastq files and bigwig files
Genome browser session<br>(e.g. UCSC)	N/A

Methodology

Replicates	2 biological replicates were performed for each experiment
Sequencing depth	at least 20 million reads per sample
Antibodies	mouse anti-FLAG M2 antibody (Sigma F1804) and Guinea pig anti-Dll antibody (Mann lab)
Peak calling parameters	MACS2 was used, with the following parameters: --nomodel –extsize 200
Data quality	Depending on experiments, from ~4000 to ~8000 peaks were called with 5% FDR cutoff. Fold enrichment varies depending on the factor being investigated.
Software	All analyses were performed using usegalaxy.org. Bowtie and MACS2 were used.
